# Eye Contact Is Crucial for Referential Communication in Pet Dogs

**DOI:** 10.1371/journal.pone.0162161

**Published:** 2016-09-14

**Authors:** Carine Savalli, Briseida Resende, Florence Gaunet

**Affiliations:** 1 Department of Public Policies and Collective Health, Federal University of São Paulo, Santos, São Paulo, Brazil; 2 Department of Experimental Psychology, University of São Paulo, São Paulo, São Paulo, Brazil; 3 Laboratoire de Psychologie Cognitive, Aix-Marseille Université, Marseille, France; University of Sussex, UNITED KINGDOM

## Abstract

Dogs discriminate human direction of attention cues, such as body, gaze, head and eye orientation, in several circumstances. Eye contact particularly seems to provide information on human readiness to communicate; when there is such an ostensive cue, dogs tend to follow human communicative gestures more often. However, little is known about how such cues influence the production of communicative signals (e.g. gaze alternation and sustained gaze) in dogs. In the current study, in order to get an unreachable food, dogs needed to communicate with their owners in several conditions that differ according to the direction of owners’ visual cues, namely gaze, head, eyes, and availability to make eye contact. Results provided evidence that pet dogs did not rely on details of owners’ direction of visual attention. Instead, they relied on the whole combination of visual cues and especially on the owners’ availability to make eye contact. Dogs increased visual communicative behaviors when they established eye contact with their owners, a different strategy compared to apes and baboons, that intensify vocalizations and gestures when human is not visually attending. The difference in strategy is possibly due to distinct status: domesticated *vs* wild. Results are discussed taking into account the ecological relevance of the task since pet dogs live in human environment and face similar situations on a daily basis during their lives.

## Introduction

A number of studies have already shown dogs’ ability to distinguish human direction of attention cues, such as body, head and eye orientation in several situations [1–4]. However, how these cues affect the production of communicative signals in dogs has not been thoroughly explored [5–7]. New information on this matter has been found and is presented here along with comparative literature and a discussion regarding its ecological relevance.

Perceiving one’s direction of visual attention is an important aspect of human development because it is related to joint attention, joint goals and the capacity of attributing mental states to others [8–10]. It is also ecologically relevant for non-human primates living in social groups, showing cooperation, sharing food and socially learning complex tasks [11–15], as well as for animal-human interactions. Furthermore, it is an important skill for communicative purposes: wild chimpanzees (*Pan paniscus*) use gesture in a functionally intentional manner adjusting to audience [16,17].

Attempts to identify which human attention cues are used by monkeys and apes in communicative contexts have led to mixed results. Rhesus macaques *(Macaca mulatta*) discriminated gross cues including body and face orientation, but not eyes status [18]. They tended to use more gaze alternation when the experimenter also displayed gaze alternation when compared to inattentive conditions. However, they did not produce vocalizations when the experimenter was inattentive. On the other hand, chimpanzees and baboons shifted from visual to acoustic communication when the recipient was not visually attending, but while chimpanzees use vocalization as means of recruiting the human’s attention, baboons use predominantly gestural acoustic signals, not vocal [19–22]. Bourjade and colleagues [19] claimed that baboons also understood the state of human eyes. In this study, food was in the experimenter’s hand and subjects seemed to distinguish more subtle cues when begging for food (open versus closed state of the experimenter’s eyes). This finding corroborates a study [11] in which capuchin monkeys successfully adjusted their requesting gestures to the human direction of attention when the experimenter was holding food, but did not discriminate these cues when they had to produce gestures directed to food on a table.

It is important to note that monkeys and apes tested in captivity usually perform tasks with no or little ecological relevance, while dogs and infants are embedded in a meaningful context, suitable to their phylogenetic and ontogenetic history. Development is always an essential issue to be considered in comparative studies of behavior [23]. Even if the ability to use human social cues was one of the traits selected during dogs’ domestication [24], it is necessary to keep in mind that “new structures and activity patterns emerge from existing structures and patterns” as stated by Lehrman in 1953 [25]. Since studies have shown that different methodologies can lead to different results, the ecological relevance of the task must be taken into account when interpreting these results.

It is widely accepted that the experimental paradigm in which dogs request for food replicates an ecological relevant situation common in their development, eliciting communicative behaviors functionally similar to those used by infants [7,26], thus representing a valid paradigm to evaluate which human attention cues are used by dogs when communicating.

There is evidence that dogs use human direction of attention when begging or performing forbidden actions [1–4]. They seem to be more sensitive to the direction of the human body and head rather than the eyes [1,27]. However, a recent study found evidence that dogs can discriminate isolated internal features of a human face, and that the eyes region seems to have a special role in human face processing [28].

Several studies in dogs showed that they are able to distinguish what humans can and cannot see in different situations, such as when they have the opportunity to steal food, to fetch toys or to beg for food [29–31]. Gaunet and Deputte [32] also found that dogs positioned themselves in an optimal location so that owners could see them when they were communicating about a target in the environment. However, dogs did not modulate communicative behaviors when they saw their owners witnessing or not a toy being hidden [26], and they hardly differentiated the eyes status of blind or sighted owners [5,33]. Moreover, they did not try to hide their approach to a forbidden food when they could not see a human present [34].

The role of experience in dogs’ ability to use human attention cues was emphasized by Udell and colleagues [3]. These authors showed that dogs chose preferentially to beg for food from an attentive person when the inattentive person was reading a book than when the inattentive person had a strange bucket over their head, which suggests that dogs were more sensitive to stimuli found in the home environment.

The ability to follow one’s gaze is also associated to visual direction [35]. In fact, a number of studies have shown that dogs are able to follow human gaze to locate hidden food [36–39]. One particular study found that dogs did not follow human gaze into a free space [40] but, according to Téglás and colleagues [39], they were more likely to do it when preceded by ostensive cues such as eye contact and addressing.

Some still argue that dogs usually avoid eye contact when fearful [41], but there are evidences that, in a friendly and cooperative situation between dogs and owners, eye contact does not pose a threat, but instead, it would facilitate communication [42]. In fact, when humans communicate with dogs, they generally use visual signals that provide information regarding the focus of attention; a recent study found that dogs displayed more attention-getting behaviors when the owners were gazing directly at them than when the owners averted their gazes [43]. Notably, eye contact signalizes for both sender and recipient that they are motivated for initializing or maintaining communication [24,44]. Moreover, Nagasawa and colleagues [45] have recently shown empirical support to the hypothesis that eye contact is involved in social attachment between dogs and owners.

Regarding the production of communicative signals, dogs use gaze alternation, sustained gaze, their own location, but little vocalization and contact, to direct the owners’ attention to a specific place [7,32,46], and this repertoire is under effect of experience [47].

Referential visual communication, as deictic behaviors and gaze following, requires joint visual attention, i.e. both sender and recipient looking towards the same referent in the environment [20,48]. According to Topál and colleagues [44], when a communicative signal directed to a referent is preceded or accompanied by ostensive cues, such as verbal addressing and eye contact, it provides indications about the sender’s intention to communicate. Bates and colleagues [49] suggested that intentional communication requires the sender to discriminate and react properly according to the recipient’s attentional state or direction of visual attention as well as availability to receive the message.

In a recent study, dogs were submitted to a naturalistic situation in which they needed to communicate with their owners in order to get an inaccessible food. Results showed that dogs met the criteria for referential and intentional communication [7]. The effect of the owners’ direction of attention on dogs’ communicative signals was analyzed taking into account body direction only. Dogs alternated more gazes between the food and their owners in the facing condition compared to when their owners had their backs turned. In another study, when dogs and toddlers were submitted to an unsolvable task situation to reach a food/toy locked in a container, they took into account body direction (facing *vs*. back turned) of the audience when exhibiting referential signals [6]. However, as far as we know no study evaluated the use of more subtle cues of visual direction of attention by dogs during such a task.

**Fig 1:**
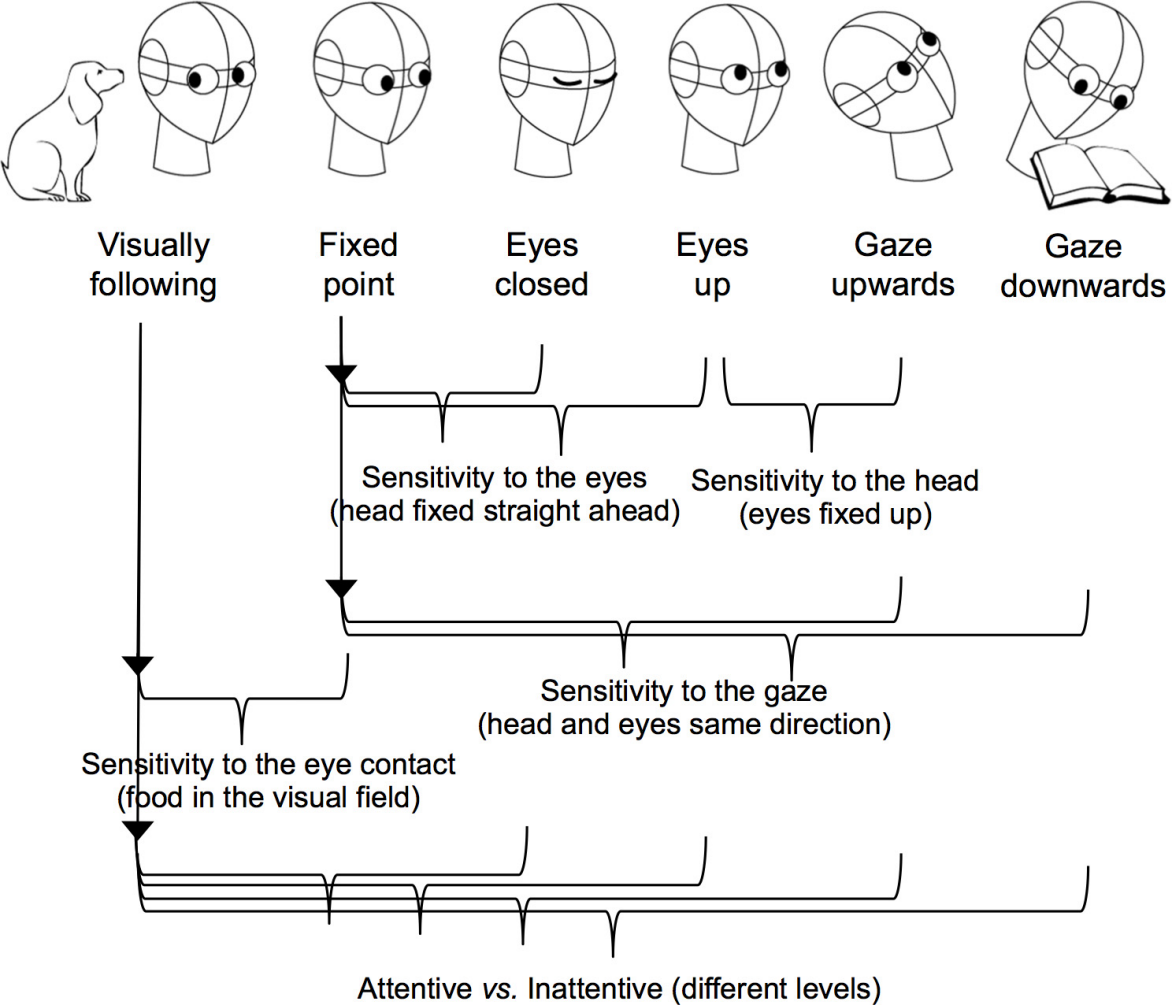
Comparisons among conditions.

The current research aimed at investigating how subtle cues of human attention (direction of eyes, head, gaze, and eye contact) affect dogs’ production of communicative signals in a food-requesting situation. For the present study, the owner was always positioned in front of the food setting. In one condition, the owner was visually following the dog and remained available to make eye contact (*Visually following*). In other five experimental conditions, the owner presented different levels of visual attention: looking straight ahead at a spot marked on the exit door (*Fixed point*), with eyes closed (*Eyes closed*), looking up by moving only the eyes (*Eyes up*), gazing upwards by moving both head and eyes (*Gazing upwards*), or reading a book (*Gazing downwards*) (Fig 1).

Since Savalli and colleagues [7] found that when the owner had their back turned to the dog, the use of gaze alternation decreased, in the current study dogs were expected to display more visual communicative behaviors such as sustained gazes (towards owner or food) and gaze alternation between the owner and the food in *Visually following* when compared to other conditions. Moreover, appropriate comparisons among conditions (Fig 1) allowed evaluating the influence of each attention cue on dogs’ behaviors and exploring how they use these subtle cues in everyday life [50].

Dogs and humans have developed a cooperative relationship throughout phylogenetic and ontogenetic history, which enabled dogs to acquire human-like communication modes such as mutual gaze [24]. Non-human primates and humans, on the other hand, have not shared such common history, except for enculturated apes. Since requesting for food replicates an everyday life experience for dogs, we expect to find in the present study more robust and clear results than those observed with non-human primates usually submitted to experimental tasks with little ecological relevance.

## General Methods

### Subjects

Twelve male and ten female adult pet dogs took part in the study (average age 5.91 (SD 2.87) years-old, minimum age 2 years-old); among them there were 7 mongrels and 15 pure breed dogs (4 Labrador Retrievers, 5 Golden Retrievers, 2 Border Collies, 1 Cocker Spaniel, 1 Beagle, 1 Pitbull, 1 Schnauzer). According to their owners, these dogs usually displayed begging behavior in the presence of food and did not present signs of distress in unfamiliar places. Owners also provided information about their dogs’ favorite food (used during experimentation) and were instructed not to feed the animals five to six hours before the trials. None of the owners wore glasses.

### Ethics Statement

This study was approved by the Committee for Ethical Research in Animals of the Institute of Psychology of University of São Paulo (process number 004.2012). Owners consented to their dogs’ participation in this study.

### Experimental Settings

**Fig 2:**
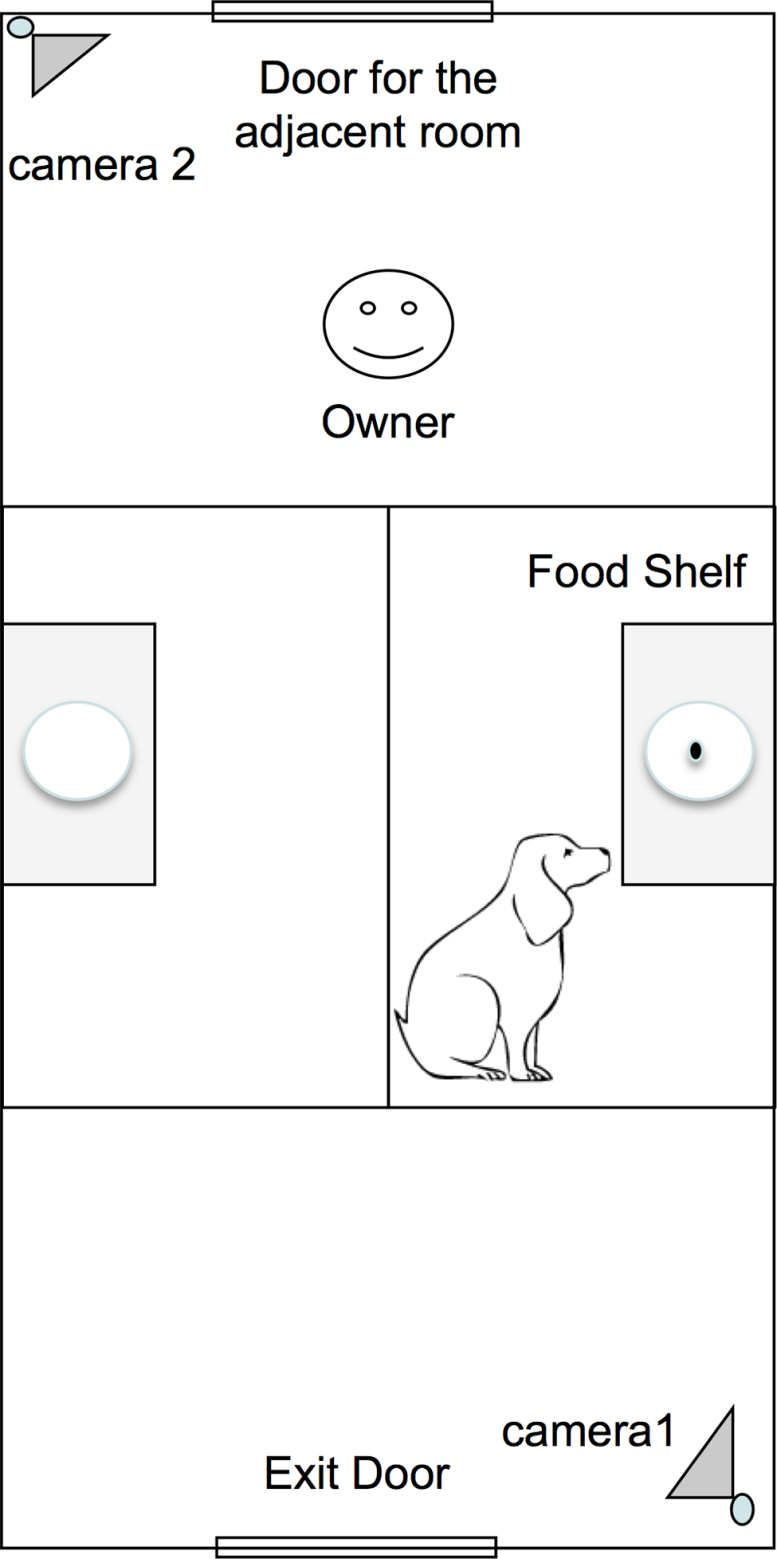
Experimental setting.

The experimental room is shown in Fig 2. There were two shelves, and in one of them (named food shelf) the dog’s favorite food was placed (the food shelf was randomly alternated across six trials for each dog). Shelves could be positioned at two different heights to make food unreachable for dogs of different sizes. The subjects could see the food and place their paws on the shelves if they stretched their bodies, but the food remained inaccessible. There was a unidirectional microphone attached to a crossbar (connecting two walls) placed in the center of the room. Two cameras recorded all trials.

### Familiarization Phase

We used a familiarization phase to show the dog that, although the experimenter was the one placing the food on the shelf, the owner would be the food provider. The owner stood at the marked location and the experimenter stood in front of the owner on the other side of the room. The experimenter placed the food on one of the shelves and returned to their location. Then the owner immediately called the dog by its name, walked towards the food shelf, got the food and gave it to the subject. This procedure was repeated alternating between the two shelves until the dog looked at the owner for the first time, as soon as the experimenter had placed the food on the shelf. The average number of repetitions required to meet this criterion was on average 8.3 (SD 2.8) times, ranging from 5 to 16 times. Therefore, the familiarization phase established a baseline for the main procedure regarding the link between the food and the owner as the food provider.

### Experimental Phase

Prior to testing, the owner received a brief explanation of experimental conditions. The experimental phase consisted of six conditions (described below) presented sequentially, only once to each dog, with intervals of approximately five minutes between conditions; the order of these six conditions was counterbalanced across dogs using block randomization. The side on which the food was positioned (left or right) was also randomized, with the restriction that one side could not be used more than twice consecutively.

A helper remained in the adjacent room, controlling entries of the experimenter, the owner and the dog into the experimental room.

Each trial included two parts: placement of food and dog exposure. In the first part, the experimenter took the dog to the experimental room with the food. She walked towards one of the shelves, got the dog’s attention, calling the dog by its name, put the food on the shelf and left the room through the exit door. Then the owner entered the experimental room and stood at their marked location (Fig 2) in front of the food setting and remained quiet. Immediately after the owner entered the room, the helper closed the door and the second part began. Dogs were presented, for 30 seconds, to one of the following condition (Fig 1):

- *Visually following*: the owner visually followed the dog and was always available to make eye contact;
- *Fixed point*: the owner remained with head and eyes directed straight ahead, looking at a spot marked on the exit door. The food was still in the owner’s peripheral visual field;
- *Eyes closed*: the owner remained with head straight ahead and his/her eyes were closed;
- *Eyes up*: the owner remained with head straight ahead, looking up only with his/her eyes, exposing the white sclera;
- *Gazing upwards*: the owner remained gazing upwards, with both head and eyes up;
- *Gazing downwards*: the owner remained gazing at a book he/she was holding, with head and eyes down.

At the end of each trial, the helper opened the door to the adjacent room and called back the owner and the dog. In the adjacent room, during the interval between conditions, dogs and owners interacted as usual, and owners gave their dogs a piece of food (that was not the shelf food), so they continued associating the situation with food availability.

*Visually following* is considered the “attentive” condition based on behavioral cues, meaning that the owner’s gaze was directed to the dog, while the other five conditions in which the owner’s gaze was not directed to the dog are considered the “inattentive” conditions.

### Behavioral Analysis and Coding

Data regarding multimodal (visual and acoustic) behaviors and dogs’ locations were coded using the Actogram Kronos software (Octarés Edition). For each dog, overlapping behaviors (i.e. not mutually exclusive) were defined with or without movements [7,32]:

- Gazing at owner: the dog’s head and nose were oriented towards the owner's face;
- Gazing at food: the dog’s head and nose were oriented towards the food;
- Gaze alternation between the owner and the food: gaze at the owner’s face followed by gaze at the food (or vice-versa) within 2 seconds;
- Gazing upwards: the dog’s head and nose were oriented upwards.
- Silent mouth licking: the dog displayed non-sonorous (silent) mouth licking;
- Sonorous mouth licking: the dog displayed sonorous (noisy, audible on video recording) mouth licking;
- Vocalization: the dog barked and/or whined;
- Contact with owner: the dog touched the owner with any part of its body.

Total duration of each trial (30 seconds) was used to calculate relative durations and frequencies of all behaviors, except for Gaze alternation, to which only the absolute number was used. Since behaviors could overlap (e.g. Gazing at owner and Vocalization could happen at the same time), the duration and frequency of each behavior was calculated considering all occurrences, regardless of whether it was isolated or in combination with any other behavior. In order to simplify the text, “frequency” and “duration” were used as reference to relative frequency and relative duration of variables, respectively. Regarding Gaze alternation, “number” were used as reference to absolute number.

### Location Analysis and Coding

In order to evaluate whether dogs used their own location as a local enhancement signal [32], we computed how long (duration only) subjects spent in specific areas using the location of their two front legs. Lines delimitating areas close to the shelves were painted on the floor, and the area of interest was a rectangle, 1.2 m x 1.8 m, closest to the food shelf, named Food area. Total duration of each trial (30 seconds) was also used to calculate relative duration that dogs spent in this area.

### Data Analyses

For the following variables, the duration and frequency of Gazing at owner, Gazing at food, the time spent in Food area and the number of Gaze alternation between the owner and the food, a first GEE model (Generalized Estimated Equation) was used to evaluate the demographic effects, gender and age, and the order of presentation of experimental conditions. The first three trials as well as the last three ones were pooled to test the order effect. Once established that these factors did not significantly affect target behaviors, each variable was analyzed with a GEE model including experimental condition as the fixed effect, and, the Wald statistic was used to test the specific hypotheses of this study (detailed bellow). The number of Gaze alternation was considered as a counting variable in the GEE model. The dependence caused by repeated observations in the same dog was incorporated in the model, and, a False Discovery Rate (FDR) was used to correct for multiple comparisons in the same dependent variable [51].

Vocalization, Silent mouth licking, Sonorous mouth licking, Contact with owner and Gazing upwards were rare, almost zero for all subjects in all experimental conditions (see medians and interquartile-range in S1 Table). Therefore, a non-parametric approach was adopted for them. At first, we compared all six experimental conditions using a Friedman’s test [52] for each variable. Comparisons between two conditions were performed using a Wilcoxon two-sample signed-rank tests [52], and a False Discovery Rate (FDR) was used in order to correct for multiple two by two comparisons in the same dependent variable [51].

### Hypotheses to address the dogs’ sensitivity to subtle cues of attention

#### Sensitivity to direction and visibility of eyes

In order to evidence the influence of human eyes on dogs’ communicative signals, the *Fixed point* condition was compared to *Eyes closed* and *Eyes up* conditions in terms of dogs’ behavior and location (Fig 1 - Sensitivity to the eyes). In these three conditions, the owner’s head remained straight ahead. However, during *Eyes up* and *Eyes closed* conditions, food was out of the owner’s visual field. Even though the owner was inattentive to the dog in these three experimental situations, if dogs use subtle information such as eye direction and visibility and discriminate when the food is in the owner’s peripheral visual field, they were expected to be more prone to display visual communicative signals in the *Fixed point* condition when compared to the other two conditions.

#### Sensitivity to different head directions

In order to evidence the influence of human head direction on dogs’ communicative signals, we compared *Eyes up* and *Gazing upwards* conditions in terms of dogs’ behaviors and locations (Fig 1 –Sensitivity to the head). In both conditions, the owner’s eyes remained directed up; but in the second their head was also turned upwards. Even though the owner was inattentive to the dog in both experimental situations, if dogs specifically discriminate the direction of the head, they were expected to be less prone to displaying visual communicative signals during the *Gazing upwards* condition when compared to the *Eyes up* condition.

#### Sensitivity to different gaze directions (head and eyes)

In order to evidence the influence of direction of human gaze (head and eyes) on dogs’ communicative signals, we compared the *Fixed point* condition to *Gazing upwards* and *Gazing downwards* conditions in terms of dogs’ behavior and location (Fig 1 –Sensitivity to the gaze). Even though the owner was inattentive to the dog in these three experimental situations, if dogs discriminate the direction of gaze, head and eyes together, they were expected to be more prone to displaying communicative signals during the *Fixed point* condition, when the food was in the owner’s peripheral visual field, than during the other two conditions.

#### Sensitivity to attention state and eye contact

*Visually following* represented the most favorable condition for dogs to display visual communicative signals since the owner was attentive to the dog and available to make eye contact, thus a higher rate of visual communicative signals was expected when comparing this condition to the other five experimental conditions. In order to evaluate to what extent dogs discriminate this behavioral attentive condition from different levels of owner’s behavioral inattentiveness, we compared the *Visually following* to all other conditions (Fig 1 –Attentive vs. Inattentive).

During the *Fixed point* condition, the food was in the owner’s peripheral visual field even though their head was directed straight ahead. In case of differences between this condition and the *Visually following* in terms of visual communicative behaviors, an effect of the availability of eye contact and head movement would be highlighted (Fig 1 –Sensitivity to the eye contact).

Higher differences in the rate of visual communicative signals were expected for the *Gazing upwards* and *Gazing downwards* conditions with respect to *Visually following*, since the first two were the most evident inattentive conditions.

### General information

The SAS software, version 9.2 (SAS Institute Inc., Cary, NC, USA) was used for all statistical analyses and a 5% significance level was applied. All tests were two-tailed.

A second naïve observer independently coded 36% of the sample (chosen randomly) and Kendall’s concordance coefficient (*W*) was calculated by pooling data for *Gazing downwards*, *Visually following* and *Eyes closed* conditions. Inter-observer agreement was assessed for the following variables: duration of Gazing at owner (*W* = 0.95), Gazing at food (*W* = 0.88) and the time spent in Food area (*W* = 0.98). Results indicated a good agreement between raters.

## Results

Descriptive measures were presented in S1 Table.

The first GEE model showed no gender, age or order effect for the following behaviors (df = 1 for all tests): duration of Gazing at owner (gender: Wald = 1.9, p = 0.172, age: Wald = 2.4, p = 0.123, order: Wald = 0.2, p = 0.656), frequency of Gazing at owner (gender: Wald = 3.4, p = 0.064, age: Wald = 1.9, p = 0.163, order: Wald = 0.05, p = 0.823), duration of Gazing at food (gender: Wald = 0.01, p = 0.939, age: Wald = 1, p = 0.319, order: Wald = 0.02, p = 0.885), frequency of Gazing at food (gender: Wald = 0.5, p = 0.487, age: Wald = 0.1, p = 0.756, order: Wald = 1.3, p = 0.264), the time spent in Food area (gender: Wald = 0.2, p = 0.690, age: Wald = 1.3, p = 0.249, order: Wald = 3.3, p = 0.068), and, the number of Gaze alternation (gender: Wald = 3.3, p = 0.068, age: Wald = 0.3, p = 0.590, order: Wald = 0.5, p = 0.482).

The GEE models with experimental condition as the fixed effect showed no condition effect for the frequency of Gazing at food (Wald = 4.2, df = 5, p = 0.519) and for the time spent in Food area (Wald = 7.6, df = 5, p = 0.178). However, the six conditions did differ for the following behaviors (df = 5 for all tests): Gazing at owner (duration: Wald = 37.0, p<0.001, frequency: Wald = 16.8, p = 0.005), Gazing at food (duration: Wald = 12.6, p = 0.028) and the number of Gaze alternation (Wald = 13.2, p = 0.022), therefore, these variables were analyzed regarding the specific hypotheses to address the dogs’ sensitivity to the subtle cues of human attention. The duration and frequency of Gazing at owner, the duration of Gazing at food and the number of Gaze alternation did not differ during *Fixed Point*, *Eyes up* and *Eyes closed* conditions, nor during *Eyes up* and *Gazing upwards* conditions (statistics in Table 1). The comparisons among *Fixed point*, *Gazing upwards* and *Gazing downwards* conditions (statistics in Table 1) also revealed no difference regarding those variables. Altogether, results indicated that dogs did not use specific cues from the owner’s eyes and head in order to modulate communicative behaviors and location.

**Table 1:** Comparisons for duration and frequency of Gazing at owner, duration of Gazing at food, the number of Gaze alternation using GEE models and Wald statistics (W). Significant differences are in bold. After the FDR adjustment, only the p-values shown in italics remain statistically significant.

Behavior	Comparisons	Duration	Frequency/Number
**Gazing at owner**	**Sensitivity to Direction and Visibility of Eyes**		
	*Fixed point vs*. *Eyes up vs*. *Eyes closed*	W = 0.4 (df = 2, p = 0.817)	W = 1.6 (df = 2, p = 0.446)
	**Sensitivity to Different Head Directions**		
	*Eyes up vs*. *Gazing upwards*	W = 1.1 (df = 1, p = 0.285)	W = 0.3 (df = 1, p = 0.586)
	**Sensitivity to Different Gaze Directions (Head and Eyes)**		
	*Fixed point vs*. *Gazing upwards vs*. *Gazing downwards*	W = 3.8 (df = 2, p = 0.150)	W = 1.6 (df = 2, p = 0.443)
	**Sensitivity to Attention State and Eye Contact**		
	*Visual Following vs*. *Fixed point*	W = 12.3 (df = 1**,*p = 0*.*0005***)	W = 7.2 (df = 1, ***p = 0*.*007*)**
	*Visual Following vs*. *Eyes up*	W = 2.5 (df = 1, p = 0.110)	W = 3.0 **(**df = 1, p = 0.082)
	*Visual Following vs*. *Eyes closed*	W = 3.5 **(**df = 1, p = 0.063)	W = 1.0 (df = 1, p = 0.302)
	*Visual Following vs*. *Gazing upwards*	W = 14.0 (df = 1, ***p = 0*.*0002***)	W = 5.7 **(**df = 1, ***p = 0*.*017***)
	*Visual Following vs*. *Gazing downwards*	W = 14.0 (df = 1, ***p = 0*.*0002***)	W = 11.2 (df = 1, ***p = 0*.*0008***)
**Gazing at Food**	**Sensitivity to Direction and Visibility of Eyes**		
	*Fixed point vs*. *Eyes up vs*. *Eyes closed*	W = 1.6 (df = 2, p = 0.442)	—
	**Sensitivity to Different Head Directions**		
	*Eyes up vs*. *Gazing upwards*	W = 1.1 (df = 1, p = 0.294)	—
	**Sensitivity to Different Gaze Directions (Head and Eyes)**		
	*Fixed point vs*. *Gazing upwards vs*. *Gazing downwards*	W = 5.1 (df = 2, p = 0.077)	—
	**Sensitivity to Attention State and Eye Contact**		
	*Visual Following vs*. *Fixed point*	W = 0.8 (df = 1, p = 0.380)	—
	*Visual Following vs*. *Eyes up*	W = 1.2 (df = 1, p = 0.270)	—
	*Visual Following vs*. *Eyes closed*	W = 3.8 (df = 1, p = 0.051)	—
	*Visual Following vs*. *Gazing upwards*	W = 10.9 (df = 1, ***p = 0*.*001***)	—
	*Visual Following vs*. *Gazing downwards*	W = 0.25 (df = 1, p = 0.619)	—
**Gaze alternation**	**Sensitivity to Direction and Visibility of Eyes**		
	*Fixed point vs*. *Eyes up vs*. *Eyes closed*	—	W = 1.5 (df = 2, p = 0.470)
	**Sensitivity to Different Head Directions**		
	*Eyes up vs*. *Gazing upwards*	—	W = 0.6 (df = 1, 0.443)
	**Sensitivity to Different Gaze Directions (Head and Eyes)**		
	*Fixed point vs*. *Gazing upwards vs*. *Gazing downwards*	—	W = 2.4 (df = 2, p = 0.301)
	**Sensitivity to Attention State and Eye Contact**		
	*Visual Following vs*. *Fixed point*	—	W = 0.3 (df = 1, p = 0.599)
	*Visual Following vs*. *Eyes up*	—	W = 3.2 (df = 1, p = 0.074)
	*Visual Following vs*. *Eyes closed*	—	W = 0.2 (df = 1, p = 0.632)
	*Visual Following vs*. *Gazing upwards*	—	W = 10.8 (df = 1, ***p = 0*.*001***)
	*Visual Following vs*. *Gazing downwards*	—	W = 8.3 (df = 1, ***p = 0*.*004***)

**Fig 3:**
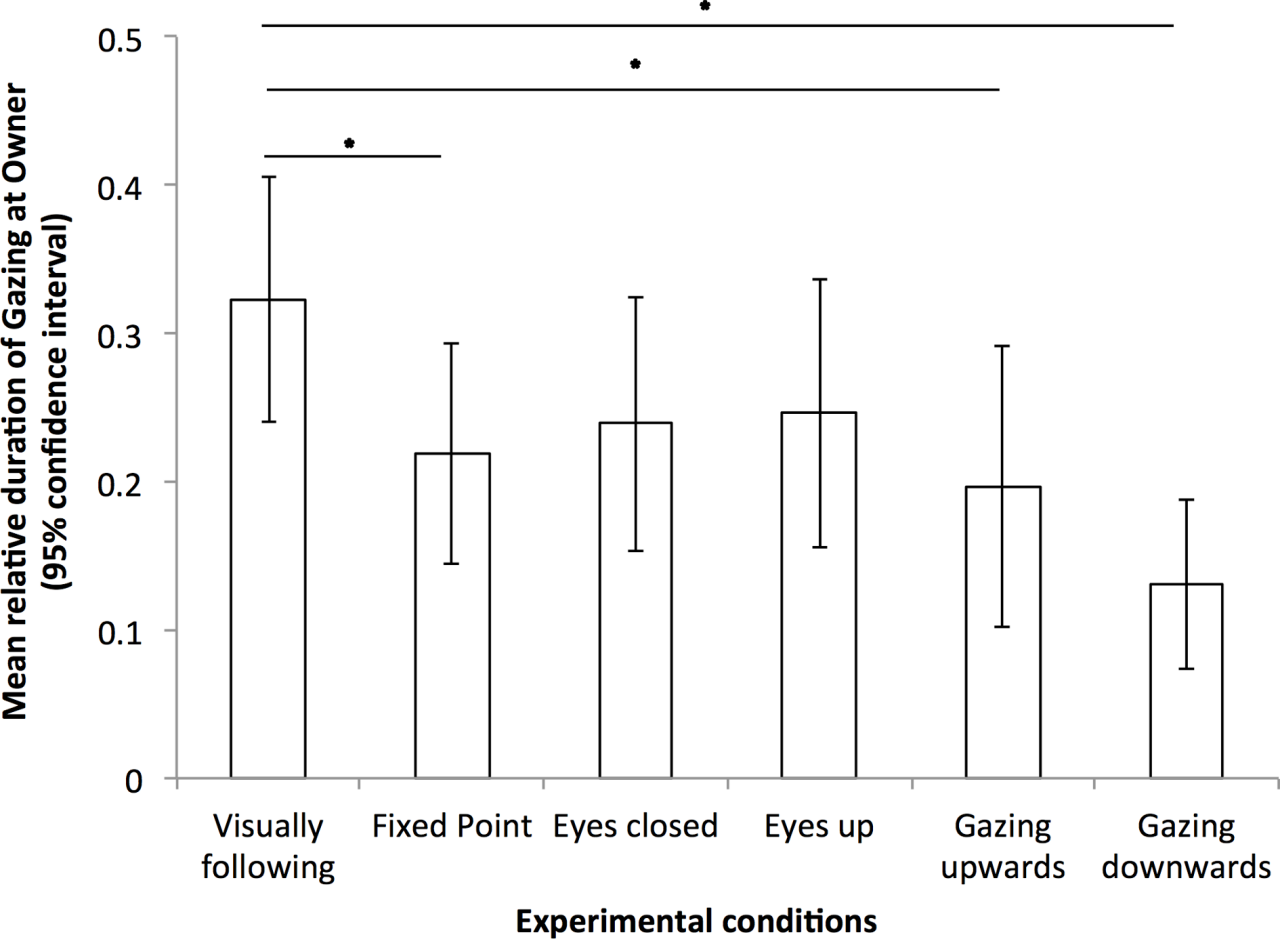
Relative duration of Gazing at owner under all six experimental conditions.

**Fig 4:**
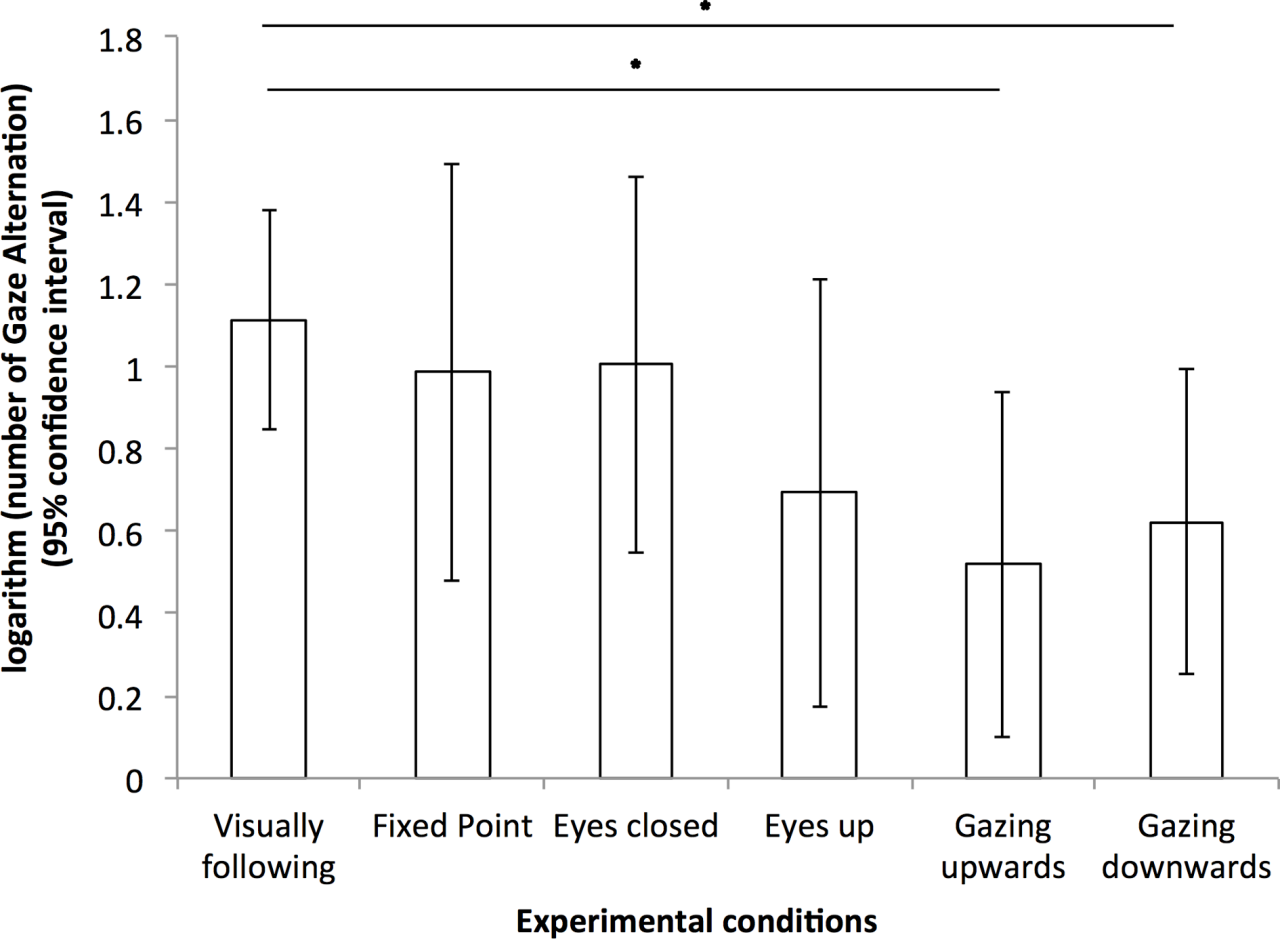
Logarithm of number of Gaze alternation under all six experimental conditions.

On the other hand, the duration and frequency of Gazing at owner were significantly greater during *Visually following* than during *Fixed point*, *Gazing upwards* and *Gazing downwards* conditions (statistics in Table 1, see Fig 3). Moreover, dogs gazed significantly longer at food during *Visually following* than during *Gaze upwards* condition. Finally, the number of Gaze alternation between the owner and the food was also significantly greater during *Visually following* than during *Gazing upwards* and *Gazing downwards* conditions (statistics in Table 1, see Fig 4).

Regarding the rare behaviors, the six conditions did not differ for (Friedman, *df = 5* for all tests): Vocalization (duration: X^2^ *=* 2.5, *p =* 0.772; frequency: X^2^ *=* 1.9, *p =* 0.858); Silent mouth licking (duration X^2^ *=* 5.9, *p =* 0.316; frequency: X^2^ *=* 4.8, *p =* 0.445); Sonorous mouth licking (duration: X^2^ *=* 0.3, *p =* 0.997, frequency: X^2^ *=* 1.7, *p =* 0.885); Contact with owner (duration: X^2^ *=* 9.6, *p =* 0.087; frequency: X^2^ *=* 9.4, *p =* 0.095). On the other hand, the six conditions did differ for Gazing upwards (duration: X^2^ *=* 17.3, *p =* 0.004, frequency: X^2^ *=* 16.9, *p =* 0.005). Therefore, the comparisons to address the dogs’ sensitivity to the subtle cues of attention were performed only for this behavior using the non-parametric approach.

The Gazing upwards did differ among *Fixed Point*, *Gazing upwards* and *Gazing downwards* conditions (statistics in Table 2): dogs tented to gaze upwards longer and more frequently when owners were also gazing upwards than when they were gazing downwards, although this difference was not significant after correction for multiple comparisons. Dogs also tended to gaze upwards more during *Gazing upwards* condition than during *Eyes up* but it was also not significant after correction for multiple comparisons. Finally, the duration of Gazing upwards was also slightly longer when owners were gazing upwards in comparison to when they were visually following dogs, although this difference was no longer significant after correction for multiple comparisons.

**Table 2:** Comparison for the duration and frequency of Gazing upwards using Friedman test and two-samples Wilcoxon rank signed tests (T). Significant differences are in bold. After the FDR adjustment, only the p-values shown in italics remain statistically significant.

Behavior	Comparisons	Duration	Frequency
**Gazing upwards**	**Sensitivity to Direction and Visibility of Eyes**		
	*Fixed point vs*. *Eyes up vs*. *Eyes closed*	χ2 = 1.6 (df = 2, p = 0.450)	χ2 = 1.6 (df = 2, p = 0.450)
	***Sensitivity to Different Head Directions***		
	*Eyes up vs*. *Gazing upwards*	T = -17 (p = **0.043**)	T = -18.5 (p = **0.031**)
	**Sensitivity to Different Gaze Directions (Head and Eyes)**		
	*Fixed point vs*. *Gazing upwards vs*. *Gazing downwards*	χ2 = 10.4 (df = 2, **p = 0.006**)	χ2 = 9.6 (df = 2, **p = 0.008**)
	*Fixed point vs*. *Gazing upwards*	T = -13.5 (p = 0.129)	T = -10.5 (p = 0.188)
	*Fixed point vs*. *Gazing downwards*	T = 3 (p = 0.250)	T = 3 (p = 0.250)
	*Gaze upwards vs*. *Gazing downwards*	T = 18 (**p = 0.031**)	T = 18.5 (**p = 0.031**)
	**Sensitivity to Attention State and Eye Contact**		
	*Visual Following vs*. *Fixed point*	T = -4 (p = 0.250)	T = -2.5 (p = 0.625)
	*Visual Following vs*. *Eyes up*	T = 0 (p = 1.000)	T = 1 (p = 1.000)
	*Visual Following vs*. *Eyes closed*	T = 0 p = 1.000)	T = 1 (p = 1.000)
	*Visual Following vs*. *Gazing upwards*	T = -21.5 (p = **0.027**)	T = -18.5 (p = 0.072)
	*Visual Following vs*. *Gazing downwards*	T = 0.5 (p = 1.000)	T = 0.5 (p = 1.000)

## Discussion

Previous studies have found that dogs adjust their communicative signals, such as gaze alternation, according to the presence of an audience and its attentional posture (cf. manipulation of the human body orientation) [6,7]. Dogs gazed longer and more frequently at their owners’ face and also alternated more gazes when owners were facing the experimental setting than when they had their back turned, supporting that the owners’ body direction, and possibly the visual access, influenced the use of referential communicative signals towards food [7]. However, no effect of the owners’ body direction on the duration and frequency of gazes at food, vocalizations or sonorous mouth licks was found.

Overall, the current study showed that dogs did not modulate communicative signals according to specific direction of attention cues such as only the owners’ eyes or only the owners’ head. On the other hand, facing impossibility to get food on their own, dogs differentiated an attentive owner, whose gaze was available to make eye contact, from an inattentive owner, gazing upwards or downwards. The two conditions in which the owner was gazing at the sky or at a book represented, in fact, the most noticeable absence of visual attention to the dog or food, since involved a changing in direction of the head and eyes altogether. This is in agreement with studies that found a differential response to the orientation of human gaze in tasks of performing an action or choosing between two unfamiliar humans to beg for food, one of them trying to make eye contact while the other one had the head turned away [1–3].

Additionally, the fact that dogs gazed significantly more at owners during *Visually following* than during *Fixed point* revealed the influence of the availability to make eye contact. However, this differential response could also be due to head movement, which is not possible to dissociate. The *Fixed point* condition can be compared to a situation in which the owner is gazing at an empty space and thus being less available to make eye contact and receive the message. This result somewhat corroborates the study of Viranyi and colleagues [2]: the verbal command “down” was less followed when the instructor was oriented to an empty space than when he was face to face with the dog. However, in the same study, dogs responded more when the instructor was gazing at an empty space than when there was another person in his visual field, as if dogs were able to distinguish when there was someone else in the instructor’s visual field that could be the recipient of that message. Based on previous findings that dogs take into account what humans can and cannot see [29,30] and that they are prone to inspect the eyes region of the human face [28], it was predicted that if dogs were able to identify that food was in their owners’ peripheral visual field, they would communicate more with their owners during the *Fixed point* than during the *Eyes up* and *Eyes closed* conditions. However, this was not confirmed in the current study. It is possible that the inspection of the eyes region was dispersed all over the face in this situation in which dogs had to divide their focus into two directions, the owner and the food, in order to make the referential communication functional. The fact that dogs did not differentiate visibility and direction of the eyes separately is in agreement with Gaunet [5,33]. In these studies, dogs from sighted owners and from blind owners behaved similarly in a communicative situation: these animals did not show sensitivity to their owners’ visual status, or, the experience of guide dogs living with blind owners did not overcome the experience of living with a sighted family during their earlier development.

In their lives, dogs have many opportunities to beg for food and it is possible that sometimes owners give them a treat even when distracted. These experiences can also mask an ability to differentiate subtle cues related to eyes visibility and direction. Particularly, the conditions during which owners had their eyes closed or up may not have been experienced by dogs and perhaps it could have elicited a strangeness or curiosity, which could also explain the tendency to continue gazing at owners under these unusual situation at the same rate than when owners visually followed the dog. As a matter of fact, dogs seem to be more prone to discriminate an attentive person from an inattentive one when the inattentive one is in a situation faced daily, such as reading a book, in comparison to a situation never experienced before [3]. Moreover, the situation in which dogs were tested in the current study also involved some level of arousal that can affect a possible ability to differentiate subtle cues regarding owners’ eyes.

Nevertheless, there is a clear distinction between discriminating different eyes directions and discriminating the availability to make eye contact, which could explain why the *Fixed point* condition did not differ from *Eyes up* and *Eyes closed*, but it did differ from the *Visually following* condition regarding the duration of Gazing at owners. It is reasonable to expect that when the owner is available to make eye contact, the dog would be encouraged to start communicating since it indicates that the person is ready to receive the message. The importance of eye contact between dogs and humans has already been shown in other contexts, e.g. dogs followed human communicative gestures more promptly when there is eye contact [24,53]. Our results highlighted, in addition, that the eye contact also elicited the production of referential communicative signals in dogs when they are in a cooperative situation.

Finally, although dogs tended to gaze at the sky longer when their owners were gazing at this direction compared to when they visually followed the dog, the effect was not confirmed after statistical correction. This result corroborates with Agnetta and colleagues [40], who indicated that dogs do not follow the human gaze into an empty space, and, with McKinley and Sambrook’s [50] who found that only two dogs out of 11 succeeded in using eye direction as a deictic cue. However, dogs followed a human gaze into the horizontal distant space when a human demonstrator used repeated gaze shifts (three consecutive sudden and fast looks to the predetermined direction for five seconds) compared to a single sudden and fast look to the predetermined direction for five seconds [54]. Therefore, inter-individual variability among dogs’ social-communicative abilities, ostensivity of the cues, training phases and early social experiences may not be ruled out as factors that influence the ability of dogs to use some human visual direction of attention cues, especially the eyes.

All in all, the present study suggests that dogs may have enhanced the referential communication, specially in terms of sustained gazes and gaze alternation that are intensified when humans provide cues that they are ready to receive the message (i.e. when dogs can rely on the whole combination of visual attention cues, including the availability to make eye contact) compared to when they are more clearly inattentive, which is ecologically relevant for dogs living in human environment. Moreover, dogs also learn about the implication of eye contact in communicative interactions on their daily life experiences [36,50,55].

Apes and baboons use vocalizations or auditory-based gestures as a way of substituting visual signals when they are not visual attending [19, 21]. On the other hand, in the current study dogs did not use vocalizations as a way to get the owner’s attention, which is in agreement with previous studies [7,32], perhaps because owners usually discourage dogs from barking and they learned to avoid this behavior regardless of the situation. It is thus likely that the ecological living conditions of these species shaped the communicative behaviors very differently, with dogs presenting more subtle/lighter behaviors than apes and baboons. The domesticated (and behaviorally shaped) *vs*. wild nature of these species may be taken into consideration when comparing their communicative behaviors.

For dogs and infants, this kind of task (interacting with a person who can provide food or a toy) is a naturalistic situation because it is similar to experiences in their everyday life environment and it is in agreement with their phylogenetic and ontogenetic history. For captive non-human primates, even though they might be habituated to human caregivers and experimenters, they were not fostered as pets and they do not have a history of domestication, which may characterize this kind of task as artificial. Captive chimpanzees, for example, are more similar to shelter dogs than to pet dogs in terms of experience with human cues, thus developmental aspects must be taken into account before assuming species differences [56]. Results vary according to each individual experience, such as housing, training and social group dynamics. Finally, small differences in methodology can have a pronounced influence on performance on these types of tasks. Artificial conditions in which monkeys and apes are submitted in captivity are probably the reason for such diverse results. Dogs’ behaviors, on the contrary, are tuned to human behaviors as a consequence of domestication and development [57]. Further comparative studies in different contexts could help to reveal the role of their particular evolutionary history, the experience and the influence of demographic factors in shaping this social-cognitive skill.

## Supporting Information

S1 Table Supplemental table 1. Descriptive measures (medians and interquartile ranges-IQR) for the relative duration and frequency of behaviors.
(DOCX)

S1 File Dataset for relative duration of behaviors and time spent in Food area. (XLSX)

S2 File Dataset for relative frequency of behaviors and number of gaze alternation. (XLSX)
