# Supplementary material for: Eye Contact Is Crucial for Referential Communication in Pet Dogs
Source: PLoS One. 2016 Sep 14;11(9):e0162161. doi: 10.1371/journal.pone.0162161 (PMC5023129; doi:10.1371/journal.pone.0162161)
Supplement: S1 Table — Descriptive measures (medians and interquartile ranges-IQR) for the relative duration and frequency of behaviors. (DOCX) [file pone.0162161.s001.docx]

**Table S1. Descriptive measures (medians and interquartile ranges-IQR) for the relative duration and frequency of behaviors.**

|  |  | **Experimental conditions** | | | | | |
| --- | --- | --- | --- | --- | --- | --- | --- |
| **Variables** |  | **Visually following** | **Fixed point** | **Eyes closed** | **Eyes up** | **Gazing upwards** | **Gazing downwards** |
| Gazing at owner | Duration | 0.30 (0.33) | 0.21 (0.29) | 0.16 (0.33) | 0.16 (0.30) | 0.11 (0.11) | 0.11 (0.09) |
|  | Frequency | 0.17 (0.10) | 0.12 (0.10) | 0.13 (0.10) | 0.13 (0.13) | 0.13 (0.13) | 0.10 (0.07) |
| Gazing at food | Duration | 0.30 (0.26) | 0.25 (0.39) | 0.14 (0.24) | 0.23 (0.25) | 0.16 (0.19) | 0.21 (0.32) |
|  | Frequency | 0.17 (0.13) | 0.13 (0.17) | 0.13 (0.07) | 0.13 (0.13) | 0.13 (0.13) | 0.15 (0.10) |
| Gaze alternations | Number | 3 (4) | 1.5 (4) | 2 (2) | 1.5 (2) | 1.5 (3) | 2 (3) |
| Vocalization | Duration | 0 (0.03) | 0 (0) | 0 (0) | 0 (0) | 0 (0) | 0 (0.02) |
|  | Frequency | 0 (0.03) | 0 (0) | 0 (0) | 0 (0) | 0 (0) | 0 (0.03) |
| Silent mouth licking | Duration | 0 (0) | 0 (0) | 0 (0) | 0 (0) | 0 (0.01) | 0 (0) |
|  | Frequency | 0 (0) | 0 (0) | 0 (0) | 0 (0) | 0 (0.03) | 0 (0) |
| Sonorous mouth licking | Duration | 0 (0) | 0 (0) | 0 (0) | 0 (0.02) | 0 (0) | 0 (0) |
|  | Frequency | 0 (0) | 0 (0) | 0 (0) | 0 (0.03) | 0 (0) | 0 (0) |
| Contact with owner | Duration | 0 (0.02) | 0 (0) | 0 (0) | 0 (0) | 0 (0) | 0 (0) |
|  | Frequency | 0 (0.03) | 0 (0) | 0 (0) | 0 (0) | 0 (0) | 0 (0) |
| Gazing upwards | Duration | 0 (0) | 0 (0) | 0 (0) | 0 (0) | 0 (0.03) | 0 (0) |
|  | Frequency | 0 (0) | 0 (0) | 0 (0) | 0 (0) | 0 (0.03) | 0 (0) |
| Food area | Duration | 0.39 (0.33) | 0.59 (0.68) | 0.23 (0.33) | 0.44 (0.40) | 0.23 (0.35) | 0.39 (0.40) |
